# Characterization of the complete chloroplast genome of *Allium tuberosum*

**DOI:** 10.1080/23802359.2019.1661302

**Published:** 2019-09-06

**Authors:** Hongxia Wang, Xiang Li, Fahui Ye, Lirong Wang, Jiuli Wang, Wenjie Chen

**Affiliations:** a Key Laboratory of Biotechnology and Analysis and Test in Qinghai-Tibet Plateau, College of Ecological Environment and Resources, Qinghai Nationalities University, Xining, China;; b Qinghai Provincial Key Laboratory of Crop Molecular Breeding, Key Laboratory of Adaptation and Evolution of Plateau Biota, Northwest Institute of Plateau Biology, Chinese Academy of Sciences, Xining, China

**Keywords:** *Allium tuberosum*, chloroplast genome, phylogenetic tree, genome engineering

## Abstract

*Allium tuberosum* is a popular vegetable, condiment, and even a traditional Chinese medicine. Here, the complete chloroplast genome sequence of *Allium tuberosum* was reported. The size of the chloroplast genome is 154,056 bp in length, including a large single copy region (LSC) of 83,068 bp, a small single copy region (SSC) of 17,958 bp, and a pair of inverted repeat (IR) regions with 26,515 bp. The *Allium tuberosum* chloroplast genome encodes 132 genes, including 87 protein-coding genes, 38 tRNA genes, and 8 rRNA genes. Phylogenetic tree analysis suggested that *Allium tuberosum* was closely related to *Allium sativum*.

*Allium tuberosum* Rottler ex Sprengel (Amaryllidaceae) is a widely cultivated perennial herb (Chung et al. 2010). It is a favorite vegetable and condiment among East Asian people. In addition, as a traditional medicinal material, *Allium tuberosum* is mainly used to treating nocturnal emissions, asthma, abdominal pain, diarrhea and sexual dysfunction (Tang et al. 2017).

Chloroplasts provide an important material basis for life on Earth through photosynthesis, and chloroplast genomes have also been smartly engineered to confer valuable agronomic traits and/or serve as bioreactors (Jin and Daniell 2015). In this study, we assembled the complete cp genome of *Allium tuberosum* (Genbank accession number: MN158715) to provide genomic and genetic sources for further research.

The fresh leaves of *Allium tuberosum* were collected from Ledu (102.33E, 36.44N), Qinghai Province, China. Total genomic DNA of *Allium tuberosum* was extracted from leaf tissues with the modified CTAB method (Doyle and Doyle 1987). The voucher specimen was deposited in Herbarium of the Northwest Institute of Plateau Biology (HNWP, WangHX2019001), Northwest Institute of Plateau Biology, Chinese Academy of Sciences. Genome sequencing was achieved on the Illumina HiSeq Platform (Illumina, San Diego, CA) at Genepioneer Biotechnologies Inc., Nanjing, China, and 8.25 GB of sequence data was generated. The trimmed reads were assembled via NOVOPlasty (Dierckxsens et al. 2017). The assembled genome was annotated using Plann v1.1 (Huang and Cronk 2015) and the annotation was corrected with Geneious v11.0.3 (Kearse et al. 2012).

The size of the complete cp genome is 154,056bp. The cp genome displayed a typical quadripartite structure, containing a pair of inverted repeated (IR) regions (26,515 bp) that divide the genome into two single-copy regions (LSC83,068 bp; SSC17,958 bp). The GC content of *Allium tuberosum* cp genome was 36.88%, with the LSC, SSC, and IR regions being 34.75%, 29.72%, 42.65%, respectively. In the *Allium tuberosum* chloroplast genome, 132 functional genes were predicted, including 87 protein-coding genes, 38 tRNA genes, and 8 rRNA genes. Most of the genes occur as a single copy in LSC or SSC, while 16tRNA genes, 8 rRNA, and 17 protein-coding genes are duplicated in the IR regions.

The maximum likelihood Neighbor-joining tree (NJ tree) was generated based on the complete cp genome of *Allium sativum* and other species of the family Orobanchaceae (Figure 1). Alignment was conducted using MAFFT (Katoh and Standley 2013).The phylogenetic tree was built using MEGA7 (Kumar et al. 2016) with bootstrap set to 10,000. The NJ tree showed that *Allium tuberosum* is closely related to *Allium sativum*. This study could lay a foundation for chloroplast genome engineering of *Allium sativum* in the future.

**Figure 1.**
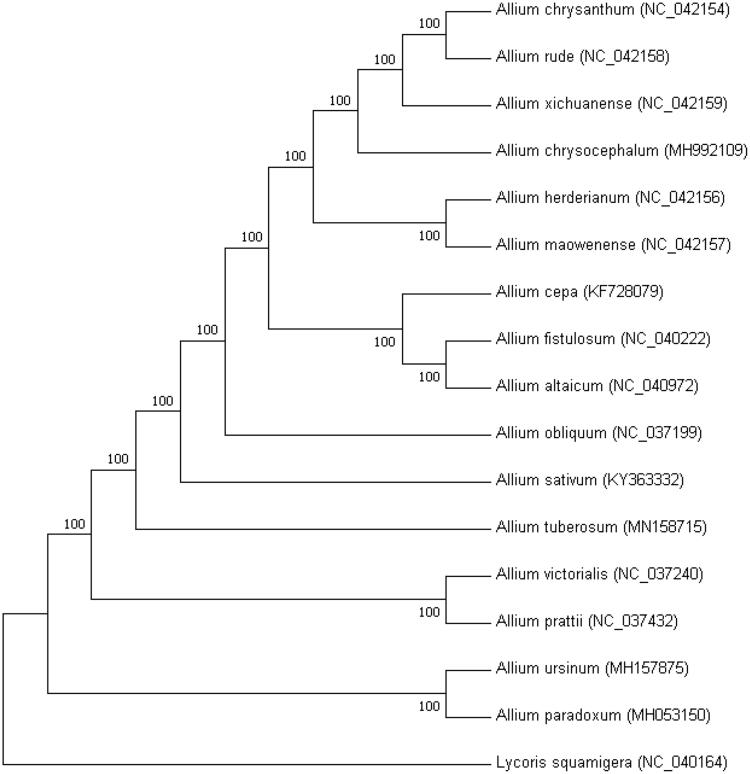
The ML tree based on 17 chloroplast genomes.
